# Dimethylaminoethyl Methacrylate/Diethylene Glycol Dimethacrylate Grafted onto Folate-Esterified Bagasse Xylan/Andrographolide Composite Nanoderivative: Synthesis, Molecular Docking and Biological Activity

**DOI:** 10.3390/molecules27185970

**Published:** 2022-09-14

**Authors:** Yue Su, Shufen Zhang, Heping Li, Bin Zhao, Kexin Tian, Zhiming Zou

**Affiliations:** 1 College of Chemistry and Bioengineering, Guilin University of Technology, Guilin 541004, China; 2 State Key Laboratory of Fine Chemicals, Dalian University of Technology, Dalian 116024, China

**Keywords:** bagasse xylan/andrographolide derivative, graft esterification, molecular docking, anticancer activity, biomass materials

## Abstract

As a biocompatible biomaterial, bagasse xylan (BX) has been widely used in the biomedical field. The low biological activity of andrographolide (AD) restricts its development, so AD with certain anticancer activity is introduced. We use chemical modification methods such as grafting and esterification to improve the biological activity and make a novel anticancer nanomaterial. On the basis of the esterification of a mixture of BX and AD with folic acid (FA), a novel anticancer nanoderivative of bagasse xylan/andrographolide folate-g-dimethylaminoethyl methacrylate (DMAEMA)/diethylene glycol dimethacrylate (DEGDMA) nanoparticles (FA-BX/AD-g-DMAEMA/DEGDMA NPs) was synthesized by introducing DMAEMA and DEGDMA monomers through a graft copolymerization and nanoprecipitation method. The effects of reaction temperature, reaction time, the initiator concentration and the mass ratio of FA-BX/AD to mixed monomers on the grafting rate (*GR*) were investigated. The structure of the obtained product was characterized by FTIR, SEM, XRD and DTG. Further, molecular docking and MTT assays were performed to understand the possible docking sites with the target proteins and the anticancer activity of the product. The results showed that the *GR* of the obtained product was 79% under the conditions of the initiator concentration 55 mmol/L, m (FA-BX/AD):m (mixed monomer) = 1:2, reaction temperature 50 °C and reaction time 5 h. The inhibition rate of FA-BX/AD-g-DMAEMA/DEGDMA NPs on human lung cancer cells (NCI-H460) can reach 39.77 ± 5.62%, which is about 7.6 times higher than that of BX. Therefore, this material may have potential applications in the development of anticancer drug or carriers and functional materials.

## 1. Introduction

Cancer is a disease with a high incidence rate and mortality, which poses a serious threat to human health [1]. According to global cancer statistics, there have been millions of new cancer cases and deaths worldwide in 2022 [2]. Although there are many methods to treat cancer, the currently used anticancer drugs have shortcomings such as toxicity, high cost and poor targeting, which seriously limit the therapeutic effect and lead to adverse side effects [3]. It remains an insurmountable global problem. Therefore, there is an urgent need to seek innovative methods.

In computer-aided drug design, molecular docking is an important method for simulating the interaction of small molecules with target proteins or for predicting their binding energy to each other [4,5]. Based on receptor–ligand interactions, molecular docking can be used to rapidly screen for effective ligands from large amounts of data, and these simulations can significantly shorten the time-consuming task of identifying potential drug candidates, thereby significantly improving the efficiency of target compound discovery [6]. To develop a therapeutic approach against COVID-19 to prevent further transmission, researchers identified three candidate agents (hispidin and lepidine E and folic acid) that inhibit the main coronavirus proteases through screening. Molecular docking revealed that all three molecules form strong hydrogen bonds with active site residues, providing a possible therapeutic strategy for COVID-19 [7]. Therefore, molecular docking simulation plays an important role in finding promising candidate drugs.

In recent years, biopolymers extracted from agricultural wastes have attracted extensive attention in the biomedical field because of their good biocompatibility and low cost [8]. In particular, xylan has shown great potential in food packaging and biomedical materials due to its good anticancer and antioxidation, making it a suitable material for drug delivery systems [9,10,11]. However, its poor solubility limits its bioavailability. In order to solve the performance defects of xylan in a single modification, we need to find a composite material with xylan to combine its properties. Recently, anticancer medicinal plants with multi-target and multi-level functions have attracted more and more interest as novel drugs, because more and more studies have found that they have potential as cancer prevention and treatment drugs [12,13]. Andrographolide (AD) is a bioactive component extracted from *Andrographis paniculata* with good anticancer activity [14]. According to the modern pharmacological studies described, andrographolide and its derivatives have attracted much attention because of their anticancer and antivirus effects [15,16]. At present, many studies are being carried out to investigate the effect of combined therapy with various anticancer drugs to overcome the limitations of known anticancer drugs [17]. It is worth mentioning that the efficacy of andrographolide combined with the anticancer drug capecitabine has been evaluated in a clinical trial for the treatment of colorectal cancer [18]. Thingale et al. proposed the compounding of andrographolide with humic acid, and they revealed that the andrographolide complex had significantly improved solubility and it also exhibited better hepatoprotection against carbon tetrachloride-induced hepatotoxicity [19]. The results showed that the composite system can effectively enhance the synergistic effect between the composites to further improve the properties and bioactivity. Given that there is no research on the composite system of andrographolide and xylan, we prepared the composite for the first time to improve the biological activities such as anticancer.

In addition, xylan is a hydrophilic polymer with extensive hydrogen bonding distributed with a large number of free hydroxyl groups along the main and side chains, which provides various possible opportunities for the modification of xylan [20]. Common modification methods include physical and chemical methods [21,22], where extensive chemical modification studies have been carried out by esterification, etherification and cross-linking to enhance its solubility and biological activity [23,24,25]. Butyrate was grafted onto xylan through esterification, and the obtained XylB increased the expression of autophagy-related proteins. The results showed that XylB may play an anti-inflammatory role in dextran sodium sulfate (DSS)-induced colitis mice [26,27]. For andrographolide, the physicochemical and pharmaceutical properties of andrographolide and its derivatives can also be improved by modifying the most accessible —OH groups on C-3, C-14 and C-19 [28]. With the application of nanotechnology in anticancer drugs, it has attracted increasing interest in cancer treatment due to its ability to improve the solubility of insoluble drugs and its passive targeting capability [29]. Using andrographolide as a nanocarrier not only can effectively improve the bioavailability of hydrophobic functional factors but also has significant advantages in enhancing their tissue distribution and antitumor activity on specific targets [30]. It was found that the solubility issue was solved by encapsulating it in poly(lactide-co-glycolide) (PLGA) nanoparticles, and the optimized formulation of Andrographis paniculata was determined to enable sustained release of the drug to improve efficacy [31]. In this experiment, andrographolide and bagasse xylan were chemically modified by esterification and graft modification, and then nanosized. On the one hand, it may push this natural compound to the forefront of human disease medicine. On the other hand, it provides new ideas for achieving effective synergistic therapy.

Although researchers have modified xylan in various ways, most of the existing research works are stuck in a single modification, which has disadvantages such as poor thermal stability and low biological activity [32,33]. In this paper, we have exploited a new folate-esterified bagasse xylan/andrographolide (BX/AD) material, which was synthesized by esterification of BX/AD and folic acid with 4-dimethylaminopyridine as a catalyst. The resulting product was obtained by introducing graft monomers via graft copolymerization. Finally, it was fabricated into nanoparticles by the nanoprecipitation method. The effects of reaction variables on the grafting rate and grafting efficiency were investigated, followed by the study of the possible action sites on protein molecules by molecular docking. The aim of the study is to synthesize functional materials with excellent bioactivity for the future, which can not only load drugs but also achieve controlled release of drugs.

## 2. Results and Discussion

### 2.1. Influences of Reaction Conditions on Grafting Rate (GR) and Grafting Efficiency (GE) of Bagasse Xylan/Andrographolide Folate-g-Dimethylaminoethyl Methacrylate/Diethylene Glycol Dimethacrylate (FA-BX/AD-g-DMAEMA/DEGDMA)

Grafting rate expressed mass percentage of the monomers (DMAEMA/DEGDMA) as grafted branches to the esterified derivative of BX/AD. Grafting efficiency indicated mass percentage of the monomers (DMAEMA/DEGDMA) as grafted branches to the total amount of the monomer.

#### 2.1.1. Influence of Reaction Temperature on GR and GE

**Figure 1:**
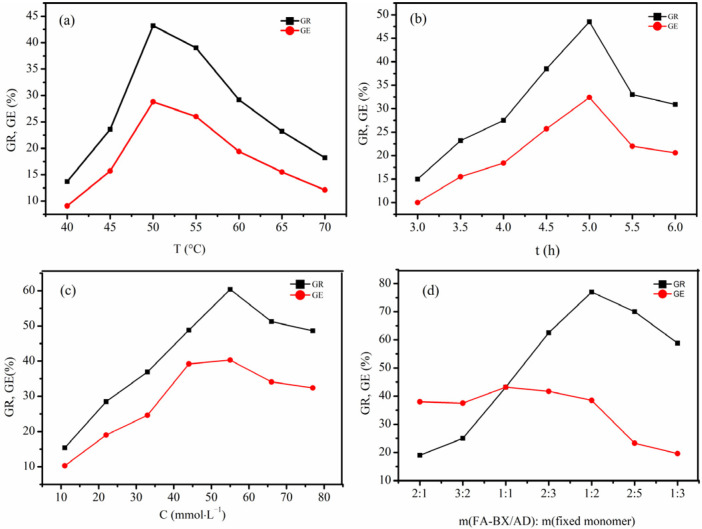
(**a**) Influence of reaction temperature on *GR* and *GE*. (**b**) Influence of reaction time on *GR* and *GE*. (**c**) Influence of initiator concentration on *GR* and *GE*. (**d**) Influence of the mass ratio of FA-BX/AD to mixed monomers on *GR* and *GE*.

Ammonium persulfate is a thermal decomposition initiator, so the reaction temperature has a great influence on the graft copolymerization reaction. The changes in grafting rate and grafting efficiency with the reaction temperature when the initiator concentration was 33 mmol/L, the mass ratio of FA-BX/AD to mixed monomers was 2:3 and the reaction time was 5 h can be seen in Figure 1a. With the continuous increase in the reaction temperature, the grafting rate and grafting efficiency increased sharply. When the temperature reached 50 °C, the grafting rate reached the maximum value. After that, with the increasing reaction temperature, both showed a downward trend. The mixed monomer reaction radicals initiated by ammonium persulfate increased with the increase in reaction temperature, and the solubility and reactivity of the mixed monomer in water increased. Therefore, the collision probability between free radicals and mixed monomers was increased, which eventually led to an increase in the grafting rate. It can be seen that the grafting rate decreased significantly when the temperature exceeded 50 °C. At high temperatures, the decrease in grafting rate is caused by the increase in chain transfer rate, homopolymerization rate and chain termination rate. Therefore, the temperature of the graft copolymerization reaction should be controlled at 50 °C.

#### 2.1.2. Influence of Reaction Time on GR and GE

The reaction times were, respectively, from 3 h to 6 h, and the adjacent time interval was half an hour. Meanwhile, the other three factors (the reaction temperature, the initiator concentration and the mass ratio of FA-BX/AD to mixed monomers) were fixed at 50 °C, 33 mmol/L and 2:3, respectively. The effect of reaction time on *GR* and *GE* is shown in Figure 1b. The grafting rate showed an upward trend with the prolongation of the reaction time in the early stage of the reaction. When the reaction was carried out for 5 h, both the grafting rate and grafting efficiency were at an extreme point. As the reaction continued, both showed a downward trend. It was observed from the analysis that the initiator triggered more FA-BX/AD grafting active sites at the beginning of the reaction when the concentration of mixed monomers was higher and the concentration of the graft copolymer produced by the reaction was relatively low. Therefore, the graft polymerization reaction can proceed rapidly, and the grafting rate and grafting efficiency increase rapidly with the increasing reaction time. With the extension of the reaction time, the concentration of reactants and active radicals in the reaction system decreased, space resistance and molecular self-polymerization increased and the grafting chains of the product produced a tendency to degrade. Therefore, the grafting rate and grafting efficiency decreased with the extension of the reaction time. From the experimental results, it can be seen that the ideal reaction time of the graft copolymerization reaction was 5 h.

#### 2.1.3. Influence of Initiator Concentration on GR and GE

The reaction was carried out at a reaction time of 5 h, a reaction temperature of 50 °C and a mass ratio of FA-BX/AD to mixed monomers of 2:3. The effect of initiator concentration on grafting rate and grafting efficiency was investigated, and the experimental results are shown in Figure 1c. At the early stage of the reaction, the grafting rate and grafting efficiency increased with increasing initiator concentration. When the initiator concentration reached 55 mmol/L, the grafting rate reached the maximum. It is known from the mechanism of graft copolymerization that the free radicals generated in the system increased with the increasing initiator concentration, which was conducive to the graft copolymerization of FA-BX/AD and mixed monomers. The grafting rate was also increased accordingly. However, when the initiator concentration was more than 55 mmol/L, the grafting rate showed a downward trend, indicating that the increase in the initiator concentration increased the probability of the mixed monomers initiating copolymerization to form homopolymers. At the same time, with the increase in initiator concentration, more free radicals were generated by the initiation and the rate of chain termination was increased, which led to a decrease in the grafting rate. It is better to control the initiator concentration at 55 mmol/L comprehensively.

#### 2.1.4. Influence of the Mass Ratio of FA-BX/AD to Mixed Monomers on GR and GE

When the initiator concentration was 55 mmol/L, the reaction time was 5 h, and the reaction temperature was 50 °C, the grafting rate and grafting efficiency of the grafted polymer varied with the mixed monomer concentration, as shown in Figure 1d. When the mass ratio of FA-BX/AD to mixed monomers was more than 1:1, the grafting rate and grafting efficiency increased with the increase in monomer concentration. After this, a non-significant decrease in grafting efficiency was observed, while the grafting rate still increased significantly. When the mass ratio was less than 1:2, the grafting rate began to decrease. The analysis showed that the initiator produced a large number of reactive radicals, which were grafted and copolymerized with the added mixed monomers. As the reaction continued, the grafting active sites on the FA-BX/AD molecules decreased, and the homopolymerization of mixed monomer radicals prevailed. As a result, the grafting rate showed a decreasing trend. In summary, the optimal mass ratio of FA-BX/AD to mixed monomers was 1:2.

### 2.2. FTIR Analysis

**Figure 2:**
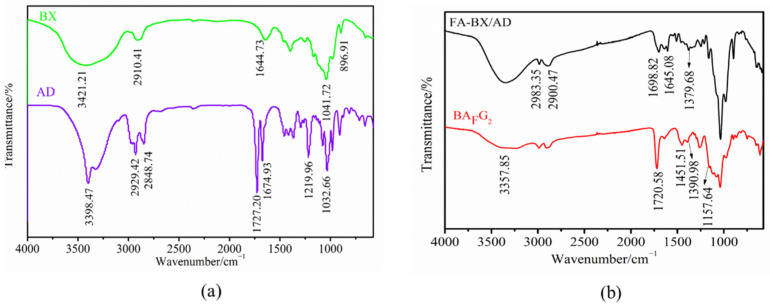
(**a**) IR spectrum of BX and AD. (**b**) IR spectrum of FA-BX/AD and BA_F_G_2_.

The IR spectra of BX and AD are shown in Figure 2a. In the IR spectrum of BX, the spectrum has a broadband at 3421.21 cm^−1^ showing strong hydrogen-bonded O—H stretching vibrational adsorption, and an absorption peak at 2910.41 cm^−1^ corresponding to the C—H stretching vibrational absorption of methyl and methylene bonds. Furthermore, the absorption peak at 1644.73 cm^−1^ was attributed to the O—H bending of adsorbed water. It can be noticed that there was a distinct band at 1041.72 cm^−1^, which can be attributed to the contribution of C—O, C—C stretch and glycosides (C—O—C). The absorption signal at 896.91 cm^−1^ was the BX molecular framework vibration peak of β-1, 4 glycosidic bond configuration. From the IR spectrum of AD in Figure 2a, it can be seen that the characteristic absorption peak around 3398.47 cm^−1^ was ascribed to the vibration absorption peak of free hydroxyl in AD. The peaks at 2929.42 cm^−1^ and 2848.74 cm^−1^ were assigned to the symmetric and asymmetric stretching vibrations of the C—H single bond, respectively. Moreover, the characteristic absorption peak around 1727.20 cm^−1^ and 1674.93 cm^−1^ corresponded to the stretching vibration of carbonyl —C=O esters. The corresponding characteristic absorption peaks near 1219.96 cm^−1^ and 1032.66 cm^−1^ were due to the antisymmetric stretching vibration and stretching vibration peaks of ester —C—O—C—, respectively.

The IR results of FA-BX/AD and FA-BX/AD-g-DMAEMA/DEGDMA (BA_F_G_2_) are shown in Figure 2b. The FTIR spectra showed that the esterification had occurred from the considerable differences between the unmodified BX and AD. In addition, it can be observed that the single absorptive band in the unmodified xylan at 2910.41 cm^−1^ was changed to double absorptive bands (2983.55 cm^−1^ and 2900.47 cm^−1^) in the modified xylan, which may be attributed to the introduction of more methylene groups (C—H bonds) from the FA. After the esterification reaction, a new absorption peak was recorded at 1698.82 cm^−1^, which was attributed to the stretching vibration of the carbonyl group (C=O). In addition, 1645.08 cm^−1^ was the stretch vibration peak of the peptide bond in folic acid and 1379.68 cm^−1^ was the bending vibration peak of methyl C—H in the folic acid molecule. The infrared spectrum of BA_F_G_2_ showed that the hydroxyl stretching vibration peak at 3357.85 cm^−1^ was significantly weakened after the introduction of grafted monomers DMAEMA and DEGDMA while keeping the characteristic peaks of FA-BX/AD unchanged. Moreover, the stretching vibration peak of ester carbonyl C=O at 1720.58 cm^−1^ was obviously enhanced. The bending vibration peak of the alkyl in DMAEMA appeared at 1451.51 cm^−1^ and the stretching vibration peak of methyl C—H in DMAEMA and DEGDMA appeared at 1390.98 cm^−1^. The peak at 1157.64 cm^−1^ corresponded to the stretching vibration peaks of ester C—O bonds in DMAEMA and DEGDMA.

In summary, it can be seen from these characteristic peaks that characteristic groups of folic acid, DMAEMA and DEGDMA were introduced into the product.

### 2.3. XRD Analysis

**Figure 3:**
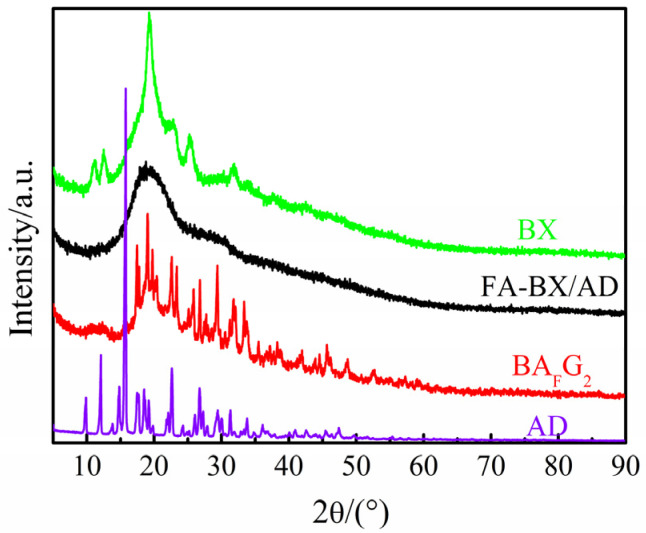
XRD profile of BX, AD, FA-BX/AD and BA_F_G_2_.

The crystallinity of BX, AD, FA-BX/AD and BA_F_G_2_ was analyzed by an X-ray diffractometer. It can be seen from Figure 3 that BX has diffraction peaks with wider peak shapes at diffraction angles of 11°, 12.5°, 19.3°, 22.8°, 25.3° and 31.7°. It indicated that BX itself had a certain crystalline region, and showed an amorphous structure and a weak peak shape. The diffraction peaks of AD appeared at diffraction angles of 9.8°, 12°, 14.7°, 15.7°, 18.5°, 19.2°, 22.6°, 26.7°, 29.4°, 31°, 33.8°, which were sharp and prominent, indicating that andrographolide had high crystallinity and a complete crystalline region. Compared with BX and AD, the X-ray diffraction spectra of FA-BX/AD had changed greatly, and the diffraction peak at the diffraction angle of 19.3° became broad and blunt. Meanwhile, the crystalline peaks in the XRD patterns of BX and AD disappeared, which illustrated that BX and AD were destroyed and their crystallinity was obviously reduced during the esterification modification. It was demonstrated that the new diffraction peaks of BA_F_G_2_ appeared at the diffraction angles of 19.7°, 23.4°, 27.7° and 45.6°, and the overall peak shape was high, narrow and concentrated when comparing the XRD patterns of BA_F_G_2_ with those of BX and AD. Moreover, the diffraction peaks of BX at 11° and 12.5° disappeared, while the diffraction peak at 31.7° became stronger. This illustrates that the crystalline content of the product increased and the crystalline region became larger after the esterification grafting cross-linking modification.

### 2.4. Derivative Thermogravimetric (DTG) Analysis

**Figure 4:**
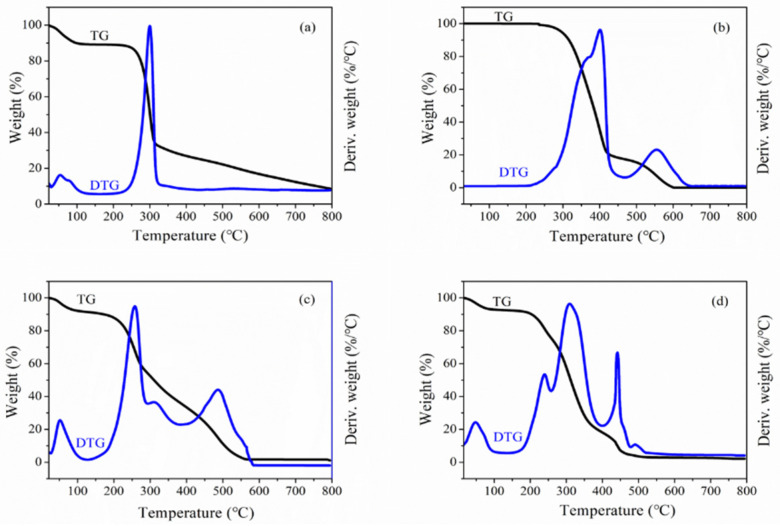
(**a**) TG and DTG curves of BX. (**b**) TG and DTG curves of AD. (**c**) TG and DTG curves of FA-BX/AD. (**d**) TG and DTG curves of BA_F_G_2_ (where the TG curve is marked in black and the DTG curve is marked in blue).

Thermogravimetric analysis was performed to understand the thermal stability of the BX and AD, and the results are shown in Figure 4a,b. It can be observed that the change in weight loss is gradual. According to the analysis, BX is reduced in three steps, while AD is reduced in two steps. The first stage where the weight of the sample starts to decrease around 100 °C was mainly due to the removal of the water existing in the sample [34]. The weight loss reduction in the second stage from 220 to 320 °C is caused by the decarboxylation, dehydration and oxidative decomposition of hemicellulose macromolecules [35]. In the temperature range from 320 to 800 °C, the mass degradation rate of BX samples was slightly lower than that of the second stage. Due to the fracture of the residue on the BX skeleton [36], the mass loss was about 25%. As for AD, its first stage was from 250 to 450 °C, which may be caused by the breaking and decomposition of the enterolactone ring and the diterpene bicyclic ring in the AD molecule. Hence, the mass decreased faster with a mass loss of 80%. The mass loss was about 20% in the range from 450 to 600 °C, which might be caused by the breaking of exocyclic double bonds in the AD molecule.

The thermogravimetric and derivative thermogravimetric (TG-DTG) curves of FA-BX/AD and BA_F_G_2_ are shown in Figure 4c,d. It can be seen from Figure 4c that the mass loss of FA-BX/AD was mainly divided into three stages in the process from 0 to 800 °C. During the process from 0 to 100 °C, the quality of the sample decreased slightly, which was mainly affected by the evaporation of moisture, residual acetone and ethanol from the sample, whose in-process mass loss was about 10%. The decrease in weight loss over the period from 200 to 380 °C was caused by the breakdown of the glycosidic bonds in the xylan backbone and the breakage of the ring-loading structure within the AD molecule [37]. The third stage was from 380 to 550 °C, and the mass loss in this stage was about 30% which might be caused by the breakage of the ester bond linking BX and AD to folic acid in the sample. From Figure 4d, it can be seen that the mass-loss process of BA_F_G_2_ varies in the interval from 0 to 800 °C, similar to FA-BX/AD, and could be divided into three stages. Their difference lies in the reason for the mass loss in the second stage, which is not only the breakage of the glycosidic bond on the BX backbone and the ring structure in the AD molecule but also the breakage of the mixed monomer side chain.

In summary, the TG-DTG analysis of the product showed that the mass loss and the thermal decomposition rate of FA-BX/AD at low temperatures were significantly lower than those of BX and AD, which indicated that the thermal stability of the esterified product was improved. The analysis of BA_F_G_2_ showed that its mass loss from 0 to 100 °C was lower than that of BX. Meanwhile, its decomposition rate in the range of 220 to 320 °C was also lower than that of BX, indicating that the thermal stability of BA_F_G_2_ was higher than that of BX at low temperatures.

### 2.5. SEM Analysis

**Figure 5:**
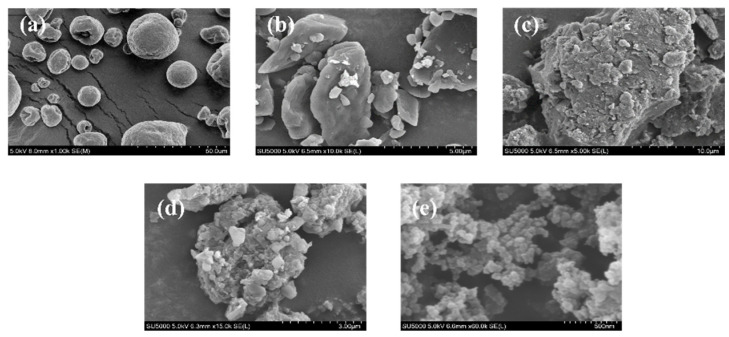
(**a**) SEM image of BX. (**b**) SEM image of AD. (**c**) SEM image of FA-BX/AD. (**d**) SEM image of BA_F_G_2_. (**e**) SEM image of BA_F_G_2_ nanoparticle.

The SEM micrographs of the surfaces of BX, AD, FA-BX/AD, FA-BX/AD-g-DMAEMA/DEGDMA (BA_F_G_2_) and FA-BX/AD-g-DMAEMA/DEGDMA (BA_F_G_2_) nanoparticles are shown in Figure 5. As shown in Figure 5a, the overall morphology of BX appeared spherical with a smooth surface and reasonable structure. At the same time, there was also a compact and highly fibrotic morphology. AD is flattened crystals of different sizes, and has a smooth surface without breakage. However, it was unevenly dispersed (Figure 5b). The surface morphology of FA-BX/AD was significantly damaged by the esterification treatment, producing a substantial number of cracks and pores (Figure 5c). The overall appearance of FA-BX/AD obtained by esterification was agglomerated, and the interstices among the particles were significantly reduced. In the meantime, its surface morphology was significantly destroyed. Not only was the surface rough, but also a large number of cracks and pores were produced. Based on the flat block-like structure, the surface of BA_F_G_2_ became more rough and porous, along with a layer of white mesh attached to it (Figure 5d). It is possible that the side chains formed by the grafted monomers were aligned on the surface of the products, indicating that the esterification graft cross-linking modification successfully introduced the folate groups and grafted monomers, so it altered the surface morphology of bagasse xylan and andrographolide. As shown in Figure 5e, the BA_F_G_2_ nanoparticles were prepared by the nanoprecipitation method, and had a spherical morphology and a particle size of about 100–200 nm.

### 2.6. Molecular Docking

**Figure 6:**
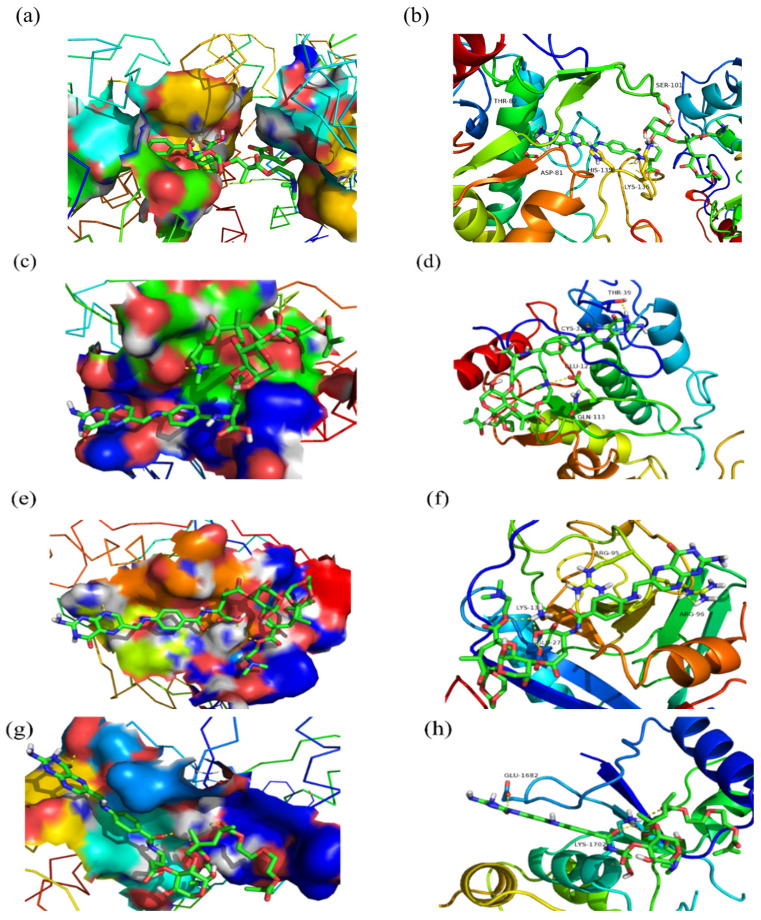
(**a**) BA_F_G_2_ and 4LRH docking conformation. (**b**) The docking site of BA_F_G_2_ within the active site of 4LRH. (**c**) BA_F_G_2_ and 4KMZ docking conformation. (**d**) The docking site of BA_F_G_2_ within the active site of 4KMZ. (**e**) BA_F_G_2_ and 2HQ6 docking conformation. (**f**) The docking site of BA_F_G_2_ within the active site of 2HQ6. (**g**) BA_F_G_2_ and 1JNX docking conformation. (**h**) The docking site of BA_F_G_2_ within the active site of 1JNX.

Computer-aided drug design utilizes computer technology to study the relationship between drug structure and biological activity at the molecular level. By designing the molecular mechanism of drugs, the success rate of drug design has been greatly improved. The interaction of BA_F_G_2_ with four receptor proteins (4LRH, 4KMZ, 2HQ6 and 1JNX) was performed by semi-flexible molecular calculations using Auto Dock software, and then the binding of the small molecule (BA_F_G_2_) to the proteins was evaluated by the magnitude of the binding energy. The docking results are shown in Table 1. The estimated free energy of binding ranges from −0.62 kcal/mol to −4.12 kcal/mol. Among them, we found that BA_F_G_2_ had the lowest binding energy with 4LRH, indicating its best docking activity. Then, Pymol was used to make the binding mode diagram of BA_F_G_2_ with four receptor proteins (4LRH, 4KMZ, 2HQ6, 1JNX), in which the receptor protein was shown in the form of a cartoon, BA_F_G_2_ was shown as a stick model and the hydrogen bond was shown as a yellow dotted line, as shown in Figure 6.

Figure 6a shows that the receptor protein 4LRH exhibits a distinct active pocket. BA_F_G_2_ was successfully embedded into the receptor protein through the active pocket, indicating the presence of a large hydrophobic cavity in which a strong hydrophobic effect exists. The amino acid residues that BA_F_G_2_ hydrogen-bonded with 4LRH are THR-82, ASP-81, HIS-135, LYS-136 and SER-101. Meanwhile, the estimated free energy of binding of BA_F_G_2_ with 4LRH is −4.12 kcal/mol. As shown in Figure 6d, the amino acid residues that BA_F_G_2_ hydrogen-bonded with 4KMZ are THR-39, CYS-31, GLU-121 and GLN-113. Molecular docking analysis showed that the estimated free binding energy of BA_F_G_2_ with 4KMZ was −0.62 kcal mol. It can be seen from Figure 6f that the estimated free energy of binding of 2HQ6 is −1.77 kcal/mol and inhibits complex formation by forming hydrogen bonds with ARG-95, ARG-96, LYS-13 and GLU-27 residues. According to Figure 6h, BA_F_G_2_ forms hydrogen bonds with GLU-1682 and LYS-1702 residues, respectively, and the estimated free energy of binding of BA_F_G_2_ with 1JNX is −0.76 kcal/mol, as shown in Table 1. The above analysis demonstrated that the binding stability of BA_F_G_2_ with receptor proteins 4LRH, 4KMZ and 2HQ6 was more stable and the docking effect was effective. Comparatively, the binding stability of BA_F_G_2_ to the receptor protein 1JNX was inferior. The results of molecular docking are generally evaluated in terms of the binding free energy. The smaller the binding energy, the higher the affinity of the small ligand (BA_F_G_2_) to the receptor protein. In summary, the best docking activity was between BA_F_G_2_ and the receptor protein 4LRH.

### 2.7. Inhibition Analysis of Tumor Cell

The inhibition ratios of BX, FA-BX/AD and FA-BX/AD-g-DMAEMA/DEGDMA NPs under different mass concentrations on the following cancer cell lines: human liver cancer cells (BEL-7407), NCI-H460 (human lung cancer cells), MGC80-3B (human gastric cancer cells) and human normal lung epithelial cells (BEAS-2B), were evaluated. The results (tested by Henan Cancer Hospital and the key laboratory of pharmaceutical chemistry and drug molecular engineering in Guangxi Normal University) are shown in Table 2.

The results (shown in Table 2) indicate that BX/AD has the potential to suppress 3.85 ± 0.61% of the growth of MGC80-3B cancer cells at concentrations equal to or higher than 50 μg/mL. However, the FA-BX/AD-g-DMAEMA/DEGDMA NPs have the potential to suppress 19.78 ± 3.25% of the growth of MGC80-3B cancer cells at low concentrations (1 µg/mL). FA-BX/AD and FA-BX/AD-g-DMAEMA/DEGDMA NPs can develop an inhibitory effect on the proliferation of MGC80-3B cancer cells. The cancer suppression increases significantly with the increase in loading more products. FA-BX/AD-g-DMAEMA/DEGDMA NPs of 100 μg/mL can inhibit 39.77 ± 5.62% of NCI-H460 cancer cells, which was about 7.6 times higher than that of BX, while the inhibitory effect on MGC80-3B cancer cells was 34.87 ± 5.11%. Although the inhibitory ratio of FA-BX/AD-g-DMAEMA/DEGDMA NPs on BEL-7407 was not as good as that of NCI-H460 and MGC80-3B, it still had strong inhibitory effects on the growth of BEL-7407 cancer cells at 31.65 ± 3.79%. Furthermore, the BX/AD and FA-BX/AD-g-DMAEMA/DEGDMA NPs were nearly non-toxic to the normal cells.

## 3. Materials and Methods

### 3.1. Materials

Xylan was extracted from bagasse by an alkali method (laboratory self-made). N, N-methylene bisacrylamide (MBA) and andrographolide (AD) were purchased from Xilong Chemical Co., Ltd. (Shantou, China) and Shanghai Jiuding Chemical Co., Ltd. (Shanghai, China), respectively. Thionyl chloride (SOCl_2_) was purchased from Luoyang Chemical Reagent Factory (Luoyang, China). Ammonium persulfate (APS) was provided by Cameo Chemical Reagent Development Center. Anhydrous alcohol and N, N-dimethyl formamide (DMF) were obtained from Kaitong Chemical Reagent Co., Ltd. (Tianjin, China). Dimethylaminoethyl methacrylate (DMAEMA), diethylene glycol dimethacrylate (DEGDMA), folic acid (FA) and 4-dimethylaminopyridine (DMAP) were purchased from McLean Biochemical Technology Co., Ltd. (Shanghai, China). These chemicals were all analytically pure and used without further purification. 

Xylan was extracted and purified from bagasse xylan by an alkaline method [38,39,40]. The bagasse xylan was dried at 100 °C for 4 h before alkaline pretreatment and then ground. Subsequently, the resulting bagasse xylan was then mixed with 8% NaOH solution (where the solid/liquid ratio of bagasse xylan to NaOH solution was 1:10 (*w*/*v*)) and the mixture was added to a four-necked round-bottom flask (250 mL) equipped with a dropping funnel, condenser and magnetic stirrer, and stirred at 60 °C for approximately 4 h. After cooling to room temperature, it was filtered and the pH was adjusted to neutral by adding 25% hydrochloric acid. Anhydrous alcohol was added to the mixture until it reached three times the filtrate to precipitate bagasse xylan and the mixture was left for 48 h. Then, it was filtered by suction and washed four times with anhydrous alcohol for purification. The dried bagasse xylan product was obtained by placing it in an oven at 60 °C for 24 h.

### 3.2. Acyl Chlorination of Folic Acid

**Figure 7:**
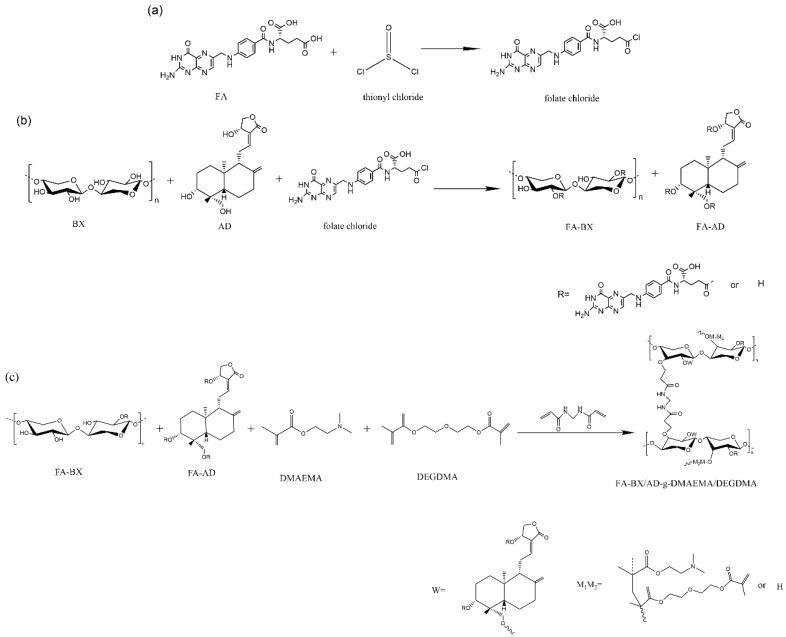
(**a**) Synthesis route of folic acid chloride. (**b**) Synthesis route of FA-BX/AD. (**c**) Synthesis route of FA-BX/AD-g-DMAEMA/DEGDMA.

We mixed folic acid (4.0 g, 9.06 mmol) and dimethyl sulfoxide (90 mL) in a 250 mL four-necked flask. The temperature of the system was controlled to 40 °C by using a collector-type constant temperature heating magnetic stirrer, and the raw material was fully stirred until dissolved. Subsequently, thionyl chloride (0.6 mL) and DMF (0.4 mL) were added to the system, and the reaction was carried out for 3 h to obtain folic acid chloride. The synthetic route of the compound is shown in Figure 7a.

### 3.3. Synthesis of Folate-Esterified BX/AD

A typical synthesis of this compound is shown in Figure 7b. When the temperature was raised to 50 °C, BX (1.34 g, 8.92 mmol) and AD (0.66 g, 1.88 mmol) were added to the flask containing the folic acid chloride (obtained by Synthesis Method 3.2), followed by DMAP (0.20 g, 1.64 mmol) as the catalyst for the esterification reaction. After continuing the reaction for 4 h, the system was cooled to room temperature and then precipitated by adding acetone (100 mL) for 30 min. Afterward, it was filtered by a vacuum pump and washed with a small amount of absolute ethanol during the process. Finally, it was placed in a constant temperature drying oven at 60 °C for 24 h to obtain FA-BX/AD, a mixture of folate-esterified BX (FA-BX) and folate-esterified AD (FA-AD). 

### 3.4. Synthesis of Folate-Esterified BX/AD-g-DMAEMA/DEGDMA

For a typical synthesis of the compound (Figure 7c), APS (0.3 g, 1.31 mmol) was added to distilled water (10 mL) until it was completely dissolved to prepare an initiator solution. According to the procedure described in Refs. [41,42,43], the graft-polymerizations of DMAEMA and DEGDMA were carried out by using the surface-initiated method, namely: DMAEMA (1.80 mL) and DEGDMA (1.84 mL) were added to a 50 mL small beaker (the mass ratio of the two grafted monomers was 1:1); then, OP-10 emulsifier (1.0 mL) and distilled water (10 mL) were added to obtain a mixed monomer solution. We mixed 2.0 g FA-BX/AD (see Figure 7b for the synthesis method) and distilled water (60 mL) in a four-necked flask and the system was stirred until completely dissolved. When the temperature increased to 60 °C, 2 mL of the above initiator solution was added. Subsequently, 1.0 mL mixed monomer solution and 1.0 mL initiator solution were added every 20 min. After the addition of the two was completed, MBA (0.2 g, 1.30 mmol) was added and the reaction was continued for 2 h. When cooled to room temperature, it was precipitated with acetone for 0.5 h, then filtered and washed with suction; it was immediately placed in a constant temperature drying oven at 60 °C for 24 h. Subsequently, the dried product was put into a Soxhlet extractor and extracted with acetone (100 mL) for 12 h, and finally dried by feeding into a constant temperature drying oven at 60 °C for 12 h to obtain FA-BX/AD-g-DMAEMA/DEGDMA (BA_F_G_2_).

### 3.5. Preparation of Folate-Esterified BX/AD-g-DMAEMA/DEGDMA Nanoparticles

First, 0.4 g FA-BX/AD-g-DMAEMA/DEGDMA was dissolved in dimethyl sulfoxide (40 mL) and stirred at 80 °C until completely dissolved. Then, it was cooled to room temperature, the solution was slowly added dropwise to anhydrous alcohol (400 mL) at a rotational speed of 800 r/min. After the dropwise addition was completed, stirring and dispersion were continued for 0.5 h. Subsequently, the solution was centrifuged at a speed of 4000 r/min and the precipitate was washed with absolute ethanol and distilled water successively, then centrifuged. Finally, the precipitate was freeze-dried to obtain FA-BX/AD-g-DMAEMA/DEGDMA nanoparticles (FA-BX/AD-g-DMAEMA/DEGDMA NPs).

### 3.6. Calculation Method for Grafting Rate (GR) and Grafting Efficiency (GE)

The degree of the graft copolymer grafting rate (*GR*) and the graft efficiency (*GE*) was calculated by the weighing method [44,45]. Then, they were respectively calculated by the Formulas (1) and (2).

(1) $$GR=\frac{W_{2}}{W_{0}}\times 100\%,$$

(2) $$GE=\frac{W_{2}}{W_{1}}\times 100\%,$$

where *GR* (%) is the grafting rate, namely the proportion of monomers involved in the grafting reaction; *W*_0_ (g) is the mass of the esterified derivative of BX/AD; *W*_1_ (g) is the total amount of the monomer before the grafting reaction; *W*_2_ (g) is the mass of the grafted branch.

### 3.7. Characterization Methods

#### 3.7.1. Fourier Transform Infrared (FTIR) Analysis

The chemical structure of samples was measured by Fourier transform infrared spectroscopy (Nicolet-iSL0, USA) in the range of 500–4000cm^−1^, and the KBr pellet technique was adopted. Each spectrum was recorded with 32 scans in transmittance mode with a resolution of 0.5 cm^−1^.

#### 3.7.2. Scanning Electron Microscopy (SEM) Analysis

The morphology characteristics of FA-BX/AD-g-DMAEMA/DEGDMA were observed by a field emission scanning electron microscope (SEM, Quanta 200 FEG, Netherlands) at different magnifications. Before the test, the dried sample was ground into powder, which was evenly coated on a conductive gel and sprayed with gold for 60 s for the scanning electron microscope test. 

#### 3.7.3. X-ray Diffraction (XRD) Analysis

The XRD patterns were collected using an X’Pert PRO (PANalytical B.V.) X-ray diffractometer equipped with Cu Kα radiation at 40 kV and 100 mA to investigate the XRD spectra of the samples. Scattered radiation was detected in the range 2θ = 5°–90°, and the speed was 10°/min.

#### 3.7.4. DTG Analysis

The thermogravimetric analysis (TG-TGA) was performed on a thermogravimetric analyzer thermogravimeter (SDT-Q600, USA) with a heating rate of 10 °C/min. All of the measurements were heated from room temperature to 800 °C under a nitrogen atmosphere to examine the thermal stability properties.

### 3.8. Molecular Docking

Protein structure-based methods were used to predict the binding conformation and binding free energy of small-molecule ligands to macromolecular targets [46]. The three-dimensional (3D) structures of FRα (gastric cancer protein, PDB Code: 4LRH), FOLR2 (lung cancer protein, PDB Code: 4KMZ), colon cancer antigen (colon cancer protein, PDB Code: 2HQ6) and BRCA1 (breast cancer protein, PDB Code: 1JNX) were obtained from the Protein Data Bank on 8th November 2021 (http://www.rcsb.org/pdb) and the small ligand (BA_F_G_2_) was energetically optimized by the Materials Studio software [47,48,49,50]. Molecular docking settings were defined using AutoDock software (version 4.2.6), which was developed and maintained by the Olson Laboratory of Scripps Research Institute (San Diego, USA).and the algorithm was executed. The protein preparation tool used to correct and optimize the structure of the PDB complex is to ensure chemical correctness by introducing the complex into Pymol to remove water molecules and organics [51,52].

### 3.9. Tumor Cell Proliferation Inhibitory Assay

Cell cytotoxicity is an important indicator for assessing the cellular health of nanomaterials for biological applications [53]. The MTT assay is one of the most commonly used colorimetric methods to assess cytotoxicity and it is also widely used to determine cytotoxicity tests for the in vitro assessment of biological materials [54]. The assay determines cell viability primarily by measuring the activity of mitochondrial enzymes, such as succinate dehydrogenase, to determine the mitochondrial function of the cell. Absorbance measurements relative to controls determine the percentage of living cancer cells remaining after treatment with different concentrations of the test compound, which is translated into the anticancer activity of the compound [55,56]. Therefore, to explore the inhibitory effect of FA-BX/AD-g-DMAEMA/DEGDMA NPs on different cancer cells, the proliferation of NCI-H460 (human lung cancer cells), MGC80-3 (human gastric cancer cells), BEL-7407 (human liver cancer cells) and BEAS-2B (human normal lung epithelial cells) was assessed via MTT assay [57,58]. The test concentrations of the materials were 1, 10, 20, 50 and 100 µg/mL, respectively. The optical density (*OD*) was measured at 490 and 630 nm, the former as a test and the latter as a reference. Blank experiments included culture fluid, MTT and DMSO. Control experiments included cells, culture fluid, MTT and DMSO. Sample experiments included materials, cells, culture fluid, MTT and DMSO. All the experiments and measurements were carried out in triplicate, and arithmetic averages were taken during the data analysis and calculations. The results were statistically analyzed using Microsoft Office Excel 2010 (Version: 14.0.4760.1000) from Microsoft Corporation (Redmond, DC, USA) and the Statistical Package for Social Sciences (version 13.0; Chicago, IL, USA). The inhibition ratio of these materials on the cancer cells was calculated by the following formula (3):

(3) $$\mathrm{Relative}\;\mathrm{Cell}\;\mathrm{Proliferation}\;\mathrm{Ratio}\;\left(RCR\%\right)=\frac{OD_{\left(\mathrm{sample},490\mathrm{nm}-630\mathrm{nm}\right)}-OD_{\left(\mathrm{blank},490\mathrm{nm}-630\mathrm{nm}\right)}}{OD_{\left(\mathrm{control},490\mathrm{nm}-630\mathrm{nm}\right)}-OD_{\left(\mathrm{blank},490\mathrm{nm}-630\mathrm{nm}\right)}}\times 100\%.$$

Inhibition ratio = 1 − *RCR*%

In our tests, the standard deviation (*SD*) was estimated and experiments were repeated to determine the experimental error. All data were reported as means  ± standard deviation. The *SD* was calculated according to the following formula (4):

(4) $$SD=\sqrt{\frac{\sum_{i=1}^{n}{\left(x_{i}-\bar{x}\right)}^{2}}{n-1}}.$$

## 4. Conclusions

In this study, the folate-esterified BX/AD derivatives with targeting properties were synthesized using BX and AD as raw materials and folic acid as an esterification agent. Then, DMAEMA and DEGDMA grafted monomers were introduced by a free radical polymerization technique. Finally, the folate-esterified BX/AD grafted nanoderivatives with the particle size of 100–200 nm were prepared by a nanoprecipitation method. The optimum process conditions for the synthesis of the target products were explored by a single factor experiment, and the target product was characterized by FTIR, SEM, XRD and TGA. Through the molecular docking simulation of the small-molecule ligand (FA-BX/AD-g-DMAEMA/DEGDMA) of the target product with the four receptor protein macromolecules, it can be seen that the best docking protein is 4LRH, which has a good affinity. Thus, it is a valuable small-ligand molecule with reasonable anticancer activity. The MTT assay showed that the novel anticancer FA-BX/AD-g-DMAEMA/DEGDMA NPs inhibited lung cancer cells (NCI-H460) by 39.77 ± 5.62% and also inhibited gastric cancer cells (MGC80-3B) by 34.87 ± 5.11%, while they were almost non-toxic to normal cells. Although the inhibitory rate of FA-BX/AD-g-DMAEMA/DEGDMA NPs on liver cancer cells (BEL-7407) was lower than that of NCI-H460 and MGC80-3B, they still had a strong inhibitory effect on the growth of BEL-7407 cancer cells.

Based on these results, it is clear that FA-BX/AD-g-DMAEMA/DEGDMA NPs have good anticancer activity and this provides a theoretical direction for the development of targeted anticancer drugs and drug carriers. The research results and methods can encourage more scholars to combine chemical modification of biomass with molecular docking to develop natural and safe antitumor drugs, drug carriers and functional materials for further exploration and research.

## Figures and Tables

**Table 1:** The dock evaluation of receptor protein and BA_F_G_2_.

PBD Code	Estimated Free Energy of Binding (kcal/mol)	Ki (μM)	Final Intermolecular Energy (kcal/mol)	Final Total Internal Energy (kcal/mol)
4LRH	−4.12	958.82	−14.56	−7.14
4KMZ	−0.62	351,290	−11.06	−6.55
2HQ6	−1.77	50,300	−12.21	−7.28
1JNX	−0.76	276,190	−11.20	−8.22

**Table 2:** The inhibition ratio of BX, BX/AD and FA-BX/AD-g-DMAEMA/DEGDMA NPs on different cancer cells and normal cells.

Sample	Mass Concentration (μg/mL)	Inhibition Ratio (%)			
		BEAS-2B	NCI-H460	MGC80-3B	BEL-7407
BX	100	1.93 ± 0.48	4.62 ± 2.79	2.02 ± 0.57	1.07 ± 0.71
	50	1.72 ± 0.76	0.71 ± 0.22	0.24 ± 0.08	1.18 ± 0.34
	20	−0.26 ± 0.57	0.24 ± 0.19	−0.15 ± 0.13	0.35 ± 0.26
	10	−2.94 ± 0.35	−2.97 ± 1.43	−2.99 ± 1.11	0.47 ± 0.29
	1	−5.61 ± 0.23	−4.33 ± 2.03	−3.27 ± 1.61	−0.45 ± 0.31
BX/AD	100	1.26 ± 0.79	2.37 ± 0.73	5.62 ± 1.43	4.28 ± 1.26
	50	0.83 ± 0.61	2.42 ± 0.81	3.85 ± 0.61	2.35 ± 0.71
	20	−1.75 ± 1.02	1.28 ± 0.65	2.08 ± 0.29	1.73 ± 0.49
	10	−5.21 ± 2.23	0.93 ± 0.34	1.67 ± 0.50	0.68 ± 0.35
	1	−7.49 ± 0.38	−0.74 ± 0.69	0.83 ± 0.26	−1.23 ± 0.84
FA-BX/AD-g- DMAEMA/DEGDMA NPs	100	3.67 ± 1.38	39.77 ± 5.62	34.87 ± 5.11	31.65 ± 3.79
	50	2.83 ± 0.67	35.82 ± 5.03	30.46 ± 3.73	27.45 ± 3.60
	20	2.13 ± 0.45	31.91 ± 3.62	27.38 ± 2.96	24.81 ± 2.75
	10	1.46 ± 0.56	28.79 ± 3.19	24.59 ± 2.58	20.29 ± 2.02
	1	0.24 ± 0.18	22.64 ± 2.33	19.78 ± 3.25	16.52 ± 2.94

## Data Availability

The data in this study are available in this paper. Additional information may be available on request from the corresponding author.

## Sample Availability

Samples of the compounds are not available from the authors.
